# Determination of Characteristics of Erythromycin Resistant *Streptococcus pneumoniae* with Preferred PCV Usage in Iran

**DOI:** 10.1371/journal.pone.0167803

**Published:** 2016-12-29

**Authors:** Malihe Talebi, Azadeh Azadegan, Javad Sadeghi, Ali Ahmadi, Mostafa Ghanei, Mohammad Katouli, Parviz Owlia, Mohammad R. Pourshafie

**Affiliations:** 1 Department of Microbiology, School of Medicine, Iran University of Medical Sciences, Tehran, Iran; 2 Department of Microbiology, School of Medicine, Tehran University of Medical Sciences, Tehran, Iran; 3 Tuberculosis Department, Pasteur Institute of Iran, Tehran, Iran; 4 Genecology Research Centre, Faculty of Science, Health, Education and Engineering, University of the Sunshine Coast, Maroochydore, Queensland, Australia; 5 Molecular Microbiology Research Center (MMRC), Shahed University, Tehran, Iran; 6 Department of Microbiology, Pasteur Institute of Iran, Tehran, Iran; Cornell University, UNITED STATES

## Abstract

Amongst 100 *Streptococcus pneumoniae* isolated from clinical cases and nasopharynx of healthy individuals, 60 erythromycin resistant strains were isolated and characterized using MLST, PFGE, transposon analysis and Quellung reaction. Most of the *S*. *pneumoniae* erythromycin resistant (80%) were found to be attributable to the *ermB*-edncoded ribosome methylase activity which differs from the dominant mechanism of macrolide resistance seen in North America.

The most predominant transposons were; Tn*1545/6003* (27%), Tn*6002* (22%), Tn*2009* (20%), Tn*2010* (17%). Number of the clinical isolates carrying Tn*2010* was more significant than the normal flora. The serotypes found were; 14 (33%), 3 (22%), 23F (15%), 19F (15%), 19A (7%), 6A (3%), 9V (3%) and 6B (2%). The most prevalent serotypes among the clinical (n = 28) and normal flora (n = 32) isolates were serotypes 14 (46%) and 3 (31%), respectively. The most prevalent vaccine serotypes amongst the clinical isolates and the healthy individuals were pneumococcal conjugate vaccines (PCV) 13 and PCV10, respectively. PFGE revealed 34 pulsotypes with 9 common and 25 single types. Significant number of the normal isolates belonged to CT5 and CT6. On the other hand, significant number of clinical isolates belonged to CT8 as compared to the normal flora isolates. MLST showed 2 dominant sequence types. ST3130 (23%) and ST180 (22%) were the most predominant sequence types in the clinical and normal isolates, respectively. There was no significant difference in other sequence types between clinical and normal flora isolates. Three polyclonal complexes including Sweden^15A^ -25, Spain^23F^-1 and Spain^9V^-3 constituted 58% of the isolates. Our results suggest that the genetic diversity and transposon distribution were high among *S*. *pneumoniae*, particularly in the isolates containing *erm*(B) and double antibiotic resistant genes (*erm/mef*). The results presented here could influence the change in the current vaccination practices in Iran which currently calls for vaccination with PCV7 or PCV10.

## Introducation

*Streptococcus pneumoniae* is a common bacterial pathogen responsible for a numerous community acquired (CA) infections especially among children and elderly [1]. The pneumococcal illness varies from self-limiting mucosal infections to life-threatening invasive pneumococcal diseases [2] and eventual appearance of antibacterial resistance strains.

Macrolides are the most frequently prescribed antibiotic among CA pneumococcal infections [3]. Macrolide-resistance (MR) in pneumococci is mediated by two major mechanisms including target site modification by methylases, encoded by *ermB* (MLS_B_ phenotype) and efflux pump mechanism mediated by *mef* (M phenotype) genes with various transposons [4–6].

A recent trend of the rise of multiple drug resistant (MDR, defined as resistance to at least 3 antimicrobial classes) clinical pneumococcal strains expressing both *erm*B and *mef* carried on Tn*2010* [similar to Tn*2009* with an additional *erm*B] has been reported [5]. These isolates are clonally disseminated and often carry resistance to macrolide, lincosamide and streptogramin B (MLS_B_) [7].

The aim of this study was to compare serotyping, antibiotic resistance, clonal diversity and the presence of genetic elements in the erythromycin resistant *S*. *pneumoniae* (ERSP) isolates obtained from the clinical cases and healthy unvaccinated PCV individuals in Tehran, Iran.

## Materials and Methods

### Sample collection and identification

A total of 100 clinical and normal flora pneumococcal isolates (50 each) were collected from hospitals and private clinical laboratories in Tehran, Iran in a two-year study (2011–2013). The collected patients’ samples were from confirmed cases of meningitis, pneumonia, or bacteremia. None of the patients or healthily subjects received any forms of PCV. The normal flora isolates were collected from healthy nasopharynx individuals who did not have any antibiotic treatments for at least 6 months preceding to the sampling and had no serious nasopharynx infections. All procedures were done in accordance with the Declaration of Helsinki (1975) amended in 2013 [8]. The study was approved by the Ethics Committee of Iran University of Medical Sciences. For privacy reason, the identity of patients and clinical laboratories form whom/where the isolates had been collected remained anonymous throughout the study. A written consent, however, was obtained from the control volunteers. Standard microbiological techniques were performed for species identification, including hemolysis, Gram staining, bile solubility and susceptibility to optochin (1μg) disc [9], and identification of isolates was confirmed by *lyt*A and *ply* genes using species-specific primers for polymerase chain reaction (PCR) [10]. Serotyping was performed using the Quellung reaction with antisera (Statens Serum Institut Copenhagen, Denmark).

### Antibiotic susceptibility tests

Disk diffusion method was performed on Mueller-Hinton agar with 5% defibrinated sheep blood according to the Clinical and Laboratory Standard Institute (CLSI) guideline [9]. Antibiotic susceptibility of all erythromycin resistant isolates was determined for oxacillin (1 μg), tetracycline (15 μg), chloramphenicol (30 μg), vancomycin (30 μg), erythromycin (15 μg) clindamycin (2 μg) linezolid (30 ug), quninupristin-dalfopristin (15 ug) and trimethoprim/sulfamethoxazole (1.25/23.75 ug). All of the antibiotic discs were purchased from Mast Diagnostics Ltd (Merseyside, UK). Minimum inhibitory concentrations (MIC) for cefotaxime, ceftriaxone and amoxicillin was used according to CLSI guidelines. Interpretative criterion for kanamycin was based on guideline given by Socie′te′ Franc, aise de Microbiologie (www.sfm.asso.fr) [11]. For erythromycin and clindamycin, the isolates with MIC ≥1μg/ml were classified as resistant. *S*. *pneumoniae* strain. ATCC 49619 was used as the quality control strain [9].

### Macrolide resistant phenotype

MR phenotype was determined by the double disk diffusion method with erythromycin and clindamycin discs (D test) to differentiate constitutive and inducible resistance phenotype [7].

Erythromycin and tetracycline resistance genes PCR amplification of MR genes including *erm*B, *mef*A/E (*mef*), *tet*M and transposon related genes were done using previously published papers [4, 12].

### Transposons detection

The presence of transposons was tested using PCR assay for Tn*916* and Tn*917* genes including *xis*, *int*, *tnd*X, *tnp*R and *tnp*A. The resistance genes related to the different transposons were Tn*2009* [*tet*M, *int*, *xis*, *mef*], Tn*6002* (*erm*B, *tet*M, *int* and *xis*), Tn*2010* (*erm*B, *tet*M, *int*, *xis*, *mef*E), Tn*3872* (*erm*B, *tet*M, *tnp*A, *tnp*R), Tn*6003*/Tn*1545* (*erm*B, *tet*M, *int*, *xis*, *aph3’*-III) and Tn*1116* (*erm*B, *tet*M) [12]. PCR with primer pair J12/J11 which amplified the region orf20 to orf19 was used to distinguish among Tn*3872*, Tn*6002*, and Tn*6003*/Tn*1545*, which yield amplicons of 0.8 kb, 3.7 kb, and 7.9 kb, respectively. All primers are listed as S1 Table.

### PFGE typing

PFGE typing of the isolates was done as described before [7]. Briefly, genomic DNA was digested with *SmaI* enzyme and electrophoresis was done with ramped pulse times beginning with 5s and ending with 20s at 6 V/cm for 23 h. The banding patterns were read by Dice analysis and clustered by the unweighted pair group method with arithmetic averages (UPGMA) using Gelcompar II version 4.0 (Applied Maths, Sint-Matenslatem, Belgium).

### MLST analysis

Multi locus sequence typing (MLST) was done using seven gene targets including *aro*E, *gdh*, *gki*, *rec*P, *spi*, *xpt*, and *ddl* [13]. The amplified products were analyzed and the clusters of related sequence types (ST) were grouped into clonal complexes (CCs) using the eBURST program (http://eburst.mlst.net/).

### Statistical analysis

Differences in the proportions among categorical variables were assessed using Fisher’s exact test or the *χ*^2^ test as appropriate. Where needed statistical analysis was performed between the patient and normal flora samples.

## Results

### Bacterial isolates

In all, 60 (60%) ERSP were isolated with 28 (47%) and 32 (53%) isolates belonging to the clinical and normal flora groups, respectively. The number of ERSP isolates (clinical and normal flora) obtained was significantly higher in the individuals with <15 (76.5%) years old than in adults) ≥15 years, *P*<0.05). The rate of ERSP isolates was higher among the invasive (75%) than non-invasive (43%) clinical isolates.

### Serotypes distribution

Eight serotypes were identified among the whole isolates. These included serotypes 14 (33%), 3 (22%), 23F (15%), 19F (15%), 19A (7%), 6A (3%), 9V (3%) and 6B (2%) (Table 1). The most prevalent serotypes among the clinical (n = 28) and normal flora (n = 32) isolates were serotypes 14 (46%) and 3 (31%), respectively. They were followed by serotypes 19F (25%) and 23F (14%) for the clinical and serotypes 3 (31%) and 14 (22%) for the normal flora isolates. Additionally, the serotype 14 was significantly higher (*P*<0.05) among the invasive (67%) than non-invasive (23%) clinical isolates. On the other hand, serotype 19F was the most prevalent serotype in the non-invasive (38%) clinical isolates. Furthermore, the results showed that the serotypes 19A (13%), 6A (6%) and 6B (3%) were found only in the normal flora which constituted 22% of the isolates (Table 1).

**Table 1:** Characteristics of clinical (invasive and non-invasive isolates) and nasopharynx normal flora isolates with respect to their serological, MLST, PFGE and transposon types.

Serotype (%^a^)	Sequence type (%^b^)	Invasive (%^c^)	Noninvasive(%^c^)	Nor. Flora (%^c^)	PFGE (%^b^)	Transposon(%^b^)
**14 (33)**	3130 (30), 63 (20), 2678 (15)	10 (77)	3 (20)	7 (22)	CT2 (16), SiT (64)	6002 (35), 1545/6003 (25), 2010 (25)
**3 (22)**	180 (100)	1 (8)	2 (13)	10 (31)	CT5 (77), CT6 (23)	3872/3872+Mega (46), 1545/6003 (31), 6002 (23)
**23F (15)**	81 (67)	1 (8)	3 (20)	5 (16)	CT9 (34), CT4 (22), CT7 (22), SiT (22)	2009 (56), 6002 (22)
**19F (15)**	3130 (89)	2 (15)	5 (33)	2 (6)	CT2 (25), CT8 (25), SiT (25)	1545/6003 (34), 6002 (22), 2010 (22)
**19A (7)**	166, 199, 271, 5004 (25 each)	0	0	4 (13)	CT3 (25), CT6 (25), SiT (50)	1545/6003 (50), 1116 (25), 2009 (25)
**6A (3)**	329, 1152 (50 each)	0	0	2 (6)	SiT (100)	2009 (50), 2010 (50)
**9V (3)**	6354, 337 (50 each)	1 (6)	0	1 (3)	SiT (100)	2009 (50), 2010 (50)
**6B (2)**	7577 (100)	0	0	1 (3)	SiT (100)	2009 (100)

Abbreviations: CT: Common type, SiT: Single type.

^a^ Percent serotype from the total number of isolates (n = 60),

^b^ percent sequence type corresponding to their respective serotype,

^c^ percent of each category concomitant with the total number of isolates in each group of subjects (invasive n = 13, noninvasive n = 15, normal flora n = 32). Sequence, PFGE and transposons types with <10% of the isolates were not included.

### Antibiotic resistance

All of the ERSP isolates were susceptible to linezolid, vancomycin, imipenem, amoxicillin, cefotaxime, ceftriaxone, and quinupristin/dalfopristin. The highest resistance was found against trimethoprim-sulfamethoxazole (93%) followed by tetracycline (85%), clindamycin (78%), chloramphenicol (48%), penicillin (28%) and kanamycin (25%). In all, 83% of the ERSP isolates were MDR (resistant to 3 or more classes).

### ERSP phenotypes

**Fig 1:**
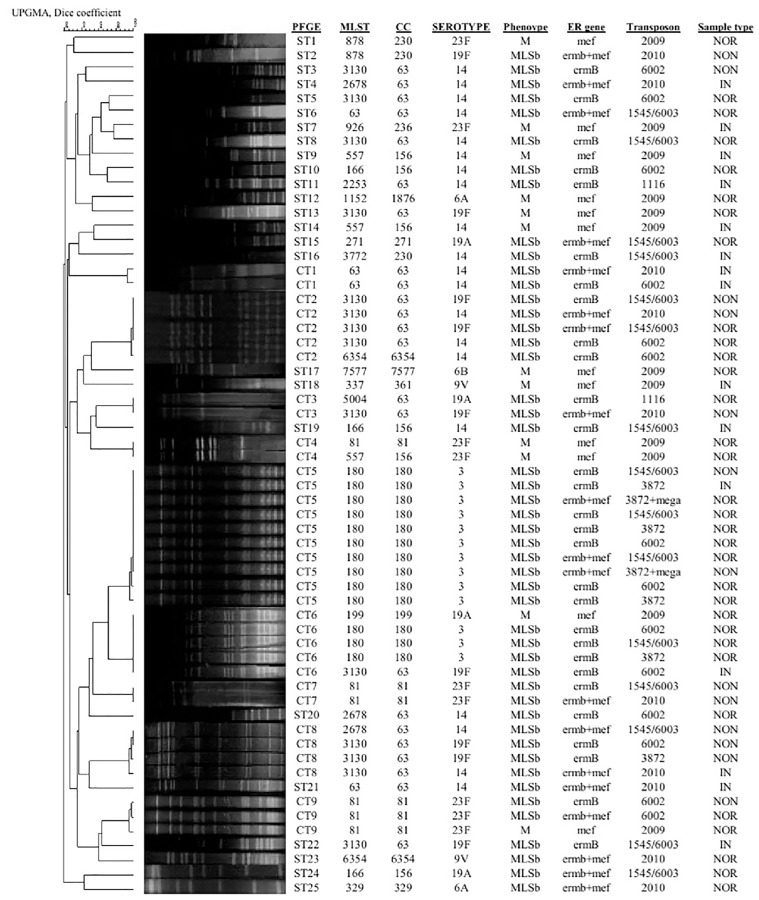
UPGMA dendrogram showing PFGE patterns of clinical (IN = invasive, NON = noninvasive) and normal flora ERSP isolates. Each pulsotype is shown in relation to the source of isolation, phenotypic and genotypic characteristics of each isolate.

On the basis of the erythromycin-clindamycin double disk test and MIC, 48 ERSP isolates were assigned to MLS_B_ phenotype (MIC >256 μg/ml) (80%) and 12 isolates were assigned to M phenotype (MIC of 1.5 to 16 μg/ml) (20%) (Fig 1). No significant difference in the distribution of MLS_B_ phenotype amongst the normal (54%) and clinical isolates (46%) was found. On the other hand, M phenotype was significantly present in the normal (67%) than the clinical isolates (33%).

### Erythromycin resistant genes distribution

No significant difference in the overall rate of erythromycin resistant genes was found between the clinical and normal flora isolates. The *erm*B, *erm*B/*mef* and *mef* were carried in 50%, 36% and 18% of the clinical isolates, respectively. The predominant double resistant genes, *erm*B/*mef*, in the clinical isolates were found in serotype 14 (60%) followed by 19F. Amongst the ERSP nasopharynx normal flora, 47%, 28% and 25% were *erm*B, *erm*B/*mef* and *mef*, respectively. In the normal flora isolates, the major serotypes carrying double resistant genes (*erm*B/*mef*) were 19A and 3 (22% each) (Fig 1).

### Transposons distribution

Amongst the total 60 ERSP isolates, Tn*1545*/Tn*6003* (27%), Tn*6002* (22%) and Tn*2009* (20%) were the most dispersed transposons (Table 2). Tn*2010* was the most common transposon in 29% of the clinical isolates followed by Tn*1545/6003* (25%). Significant differences between the clinical and normal flora isolates carrying Tn*2009* and Tn*2010* were observed. The least common type of transposons was Tn*1116* and Tn*3872*+MEGA (3% each) in both clinical and normal flora isolates (Table 2).

**Table 2:** Frequency of isolates having different transposons.

Transposon	Clinical	Normal flora	Total
**1545/6003**	7 (25%)	9 (29%)	16 (27%)
**6002**	5 (17%)	8 (25%)	13 (22%)
**2009**	4 (14%)	8 (25%)	12 (20%)
**2010**	8 (29%)	2 (6%)	10 (17%)
**3872**	2 (7%)	3 (9%)	5 (8%)
**3872+MEGA**	1 (4%)	1 (3%)	2 (3%)
**1116**	1 (4%)	1 (3%)	2 (3%)
**Total**	28 (47%)	32 (53%)	60

Percentages were calculated from the total number of ERSP isolates (n = 60)

No significant correlation was found between the type of transposon and the antibiotic resistance pattern (Table 3) in the clinical nor normal flora isolates. Resistance to kanamycin was found only in the isolates carrying Tn*1545*/Tn*6003*.

**Table 3:** Correlation between the types of transposons and their antibiotic resistance patterns in the ERSP isolates.

*Tn*	Antibiotic Resistant	Percent isolates
2009	P, TET, C	12%
2010, 6002, 1116	P, TET, CLD	18%
2010, 6002	P, C, TET, CLD	15%
1545/6003	P, KAN, TET, CLD	15%
3872, 3872+mega	C, TET, CLD	12%

All isolates were resistant to SXT. Abberviations; P, penicillin; KAN, kanamycin; CLD, clindamycin; TET, tetracycline; C, chloramphenicol. Antibiotic resistant patterns with <10% of the total isolates were not included.

### PFGE

PFGE analysis of total 60 ERSP isolates showed the presence of 34 pulsotypes with nine common types (CT) constituting 35 isolates and 25 single isolates (Fig 1). With the exception of serotype 3, no correlation was found between the serotype and PFGE type in the clinical or normal flora isolates. All serotype 3 isolates (clinical and nonclinical isolates) were found to be belonged to a single pulsotype (CT5).

### MLST

Using eBURST analysis, we identified 19 different sequence types (ST) with 10 clonal complexes (CCs) (n = 33) and 27 singletons (Figs 1 and S1). Amongst the total 60 ERSP isolates, the most prevalent STs were ST3130 and ST180 constituting 45% of all isolates followed by ST81 and ST63 (17%) (Table 4). Ten STs were found with 1 isolate each comprising 16% of the total isolates. Significant correlations between several serotypes and their STs were found. The serotypes 3, 19F and 23F showed correlations with ST180 (100%), ST3130 (89%) and ST81 (67%), respectively. On the other hand, serotype 14 isolates showed 9 different STs, the highest diversity amongst the serotypes. Furthermore, the CC63 (Sweden^15A^ -25) was the most frequent CC constituting 40% of the ERSP isolates (Table 5). Amongst the ten CCs, only CC63 and CC180 were significantly differ between the clinical and normal flora isolates (P>0.05). The results showed that all of the CC180 were serotype 3 MLS_B_ phenotype (Fig 1) which were mostly isolated from the normal flora (77%). No new alleles were found in our isolates.

**Table 4:** Distribution of MLST with their respective serotyptes.

Sequence types (%)																				
Serotype	3130	180	81	63	166	557	2678	878	6354	337	199	271	329	926	1152	2253	3772	5004	7577	Total
**14**	**6**	**0**	**0**	**4**	**2**	**2**	**3**	**0**	**1**	**0**	**0**	**0**	**0**	**0**	**0**	**1**	**1**	**0**	**0**	**20**
**19A**	**0**	**0**	**0**	**0**	**1**	**0**	**0**	**0**	**0**	**0**	**1**	**1**	**0**	**0**	**0**	**0**	**0**	**1**	**0**	**4**
**19F**	**8**	**0**	**0**	**0**	**0**	**0**	**0**	**1**	**0**	**0**	**0**	**0**	**0**	**0**	**0**	**0**	**0**	**0**	**0**	**9**
**23F**	**0**	**0**	**6**	**0**	**0**	**1**	**0**	**1**	**0**	**0**	**0**	**0**	**0**	**1**	**0**	**0**	**0**	**0**	**0**	**9**
**3**	**0**	**13**	**0**	**0**	**0**	**0**	**0**	**0**	**0**	**0**	**0**	**0**	**0**	**0**	**0**	**0**	**0**	**0**	**0**	**13**
**6A**	**0**	**0**	**0**	**0**	**0**	**0**	**0**	**0**	**0**	**0**	**0**	**0**	**1**	**0**	**1**	**0**	**0**	**0**	**0**	**2**
**6B**	**0**	**0**	**0**	**0**	**0**	**0**	**0**	**0**	**0**	**0**	**0**	**0**	**0**	**0**	**0**	**0**	**0**	**0**	**1**	**1 6**
**9V**	**0**	**0**	**0**	**0**	**0**	**0**	**0**	**0**	**1**	**1**	**0**	**0**	**0**	**0**	**0**	**0**	**0**	**0**	**0**	**2**
**T%**	**14(23)**	**13 (22)**	**6(10)**	**4 (7)**	**3 (5)**	**3(5)**	**3(5)**	**2 (3.3)**	**2(3.3)**	**1 (2)**	**1 (1.6)**	**1 (1.6)**	**1 (1.6)**	**1 (1.6)**	**1 (1.6)**	**1 (1.6)**	**1 (1.6)**	**1 (1.6)**	**1 (1.6)**	**60 (100)**

Abbreviations; S: serotype, T: total

**Table 5:** Distribution of clonal complex based on the source of isolation.

Clonal complex	Clinical	Normal flora	Total
63	15 (54%)*	8 (28%)	23 (40%)
180	3 (11%)	10 (34%)*	13 (23%)
81	3 (11%)	3 (10%)	6 (11%)
156	3 (11%)	3 (10%)	6 (11%)
230 (ST878), (ST3772)	2 (7%)	1 (33%)	3 (5%)
361 (ST337)	1 (4%)	0	1 (2%)
236 (ST926)	1 (4%)	0	1 (2%)
199	0	1 (3%)	1 (2%)
1876	0	1 (3%)	1 (2%)
271	0	1 (3%)	1 (2%)
	28	29	57

* Significantly higher (P<0.05) than the isolates obtained from the other source. Isolates with <10% were not analyzed.

## Discussion

In the present study, the overall rate of ERSP was 60% which did not significantly differ between the clinical and nasopharynx normal flora isolates. The presence of high number of MDR ERSP isolates (98%) was an indicative of indiscriminate use of antibiotics in general, and macrolide in particular, in clinical practices with eventual dissemination of the resistant strains in Iran.

Similar to the results obtained in Asia and some European countries [3, 14–16], we also found that the most common mechanism (48%) for macrolide resistance in our pneumococcal isolates was ermB-encoded ribosome methylase activity. On the contrary, efflux mediated pump gene (*mef*), which has been reported to be the dominant macrolide resistant in North American countries [17–18] and Germany [19], was found only in 20% of our isolates. Double resistant genes were present in 32% of our isolates which was significantly higher than some other investigators [20]. The prevalence of dual resistance genes in the pneumococcal isolates amongst the macrolide resistant isolates has been reported around 16% globally [17, 21] and up to 63% in China in recent years [22].

Some degree of genetic linkage was observed in this study. Our pneumococcal isolates carrying *mef* were found to be associated with 7 different serotypes (out of 8 serotypes identified), suggesting *mef* to be less discriminative than *erm*B which was found in 5 different pneumococcal serotypes (mostly in serotypes 3 and 14). Furthermore, it was observed that all isolates carrying Tn*2010* (clinical or normal flora) also harbored double resistant genes (*erm*B/*mef*). On the other hand, no association between the serotypes and transposons or the source of isolation was found. Moreover, we found that many of our isolates could carry wide range of transposons. For examples, serotype 14 in both clinical and normal flora isolates could carry all major transposons including Tn*6002*, Tn*1545/6003*, Tn*1116*, *Tn2009* and Tn*2010*. In contrary to the suggestion by Chancey and colleagues [23] that the genetic linkages of Tn*2010* with Tn*2009* and Tn*6002* may exist, we did not found any coexistence of transposons in our isolates. Table 2 shows significant difference (p<0.01) in the rate of Tn*2010* in the clinical as compared to normal flora isolates. On the other hand, significant number of the normal flora carried Tn*2009* than the clinical isolates.

Comparative analysis between the report from Asian for Surveillance of Resistant Pathogens (ANSORP) on *S*. *pneumoniae* isolated from 11 Asian countries [24], which is the most comprehensive study done so far in the Asia, with the present investigation resulted in several interesting observations. The frequency of the invasive pneumococcal isolates with 19F serotype in our and ANSORP studies was similar (13.3% vs 13.7%). The differences between ANSORP and our studies were; i) their frequency for serotype 19A was 8.2%, whereas no 19A serotype was found in our clinical isolates, ii) they found serotype 14 in 7.3% of the cases, we found in 46% of our clinical isolates (67% for the invasive isolates), iii) serotype 6A (4.2%) was the most prominent non-PCV7 serotype in the ANSORP report, whilst we observed no 6A serotype in our clinical isolates, and iv) 52% of ANSORP isolates showed PCV7 serotypes, we found significantly higher frequency (89%). These observations collectively suggest that several common *S*. *pneumococcus* serotypes have disseminated throughout the Far East, South East Asia, Middle East countries. On the other hand, some serotypes are highly localized in parts of Asian countries.

Similar to the other reports from Middle East countries [3, 25] the frequencies of our serotypes included in PCV7 and PCV10 were 89% for clinical, 50% for normal flora and 68% for total sisolates. Serotype 3 was the only non-PCV7 serotype found in our clinical study. The frequency of the serotypes included in PCV13 was 100% for both clinical and normal flora. For invasive isolates, the rates of serotypes covered by PCV7or PCV10 and PCV13 were 93% and 100%, respectively. At the present time pneumococcal conjugated vaccine is not routinely used in Iran and it is only given to a limited number of people who are at high medical risks. Our results suggested that PVC13 would be the preferred vaccine over PVC7 or PVC10 and can be implemented in the Iranian national vaccine program.

By using Pneumococcal Molecular Epidemiology Network classification,7 international resistant clones, including 19 different STs were identified among our clinical isolates. The most frequent group (23 isolates, 38%) was related to the Sweden^15A^-25 ST63 international clone with 57%, 39% and 4% harboring *erm*(B) alone, *mef* alone and *erm*B/*mef*, respectively. The second most frequent group was Spain^23F^-1 ST81 clone (6 isolates, 10%). Additionally, MLST analysis (at least six common MLST alleles out of 7) revealed nineteen STs with ST3130, ST180, ST81 and ST63 were the frequent STs covering 62% of the total cases. In a study by Calatayud and colleagues in Spain [26], 10 ST clones were reported during 1999–2007 period with Spain^23F^-1 ST81, Sweden^15A^-25 ST63 and Spain^9V^-3 ST156 constituting 30% of their invasive isolates, we found the 2 international ST clones (Sweden^15A^-25 ST63, and Spain^9V^-3 ST156) constituting 73% of our invasive isolates. On the other hand, ST81, ST283 and ST236 have been reported as the most frequent STs in some Asian countries [27, 28]. Overall, our results suggest that our pneumococcal clonal types were similar to the clones in Europe as compared to the reports in South Africa, South Korea and United States where high prevalence of Taiwan19F-14 clonal complex 271 (CC271) has been reported [20].

## Conclusions

The present study showed that the rate of resistance of *S*. *pneumoniae* strains to erythromycin was significantly high and the related isolates were clonally disseminated with majority having MLS_B_ and *ermB* phenotypic and genotypic characteristics, respectively. Additionally, we showed that within the ERSP isolates, serotypes 14, 3, 19F and 23F constituted majority (85%) of the total ERSP isolates which were belonged to ST3130 (CC63), ST180, ST3130 and ST81 clones, respectively. The data presented here suggest that the use of PVC13 could have a significant protection coverage and should be the recommended vaccine for the Iranian population living in Tehran.

## Supporting Information

S1 Fig 60 ERSP isolates as shown by eBURST analysis. One spot indicates one ST. The size of one spot relates to the number of pneumococcal isolates with the same ST. The lines designate the presence of single locus variant SLV and DLV links amongst STs.
(DOCX)

S1 Table Primers used for identification of pneumococcal isolates and amplification of erythromycin and tetracycline resistant genes as well as transposon related genes. (DOCX)
