# Supplementary material for: Determination of Characteristics of Erythromycin Resistant Streptococcus pneumoniae with Preferred PCV Usage in Iran
Source: PLoS One. 2016 Dec 29;11(12):e0167803. doi: 10.1371/journal.pone.0167803 (PMC5199012; doi:10.1371/journal.pone.0167803)

S1 Fig: 60 ERSP isolates as shown by eBURST analysis. One spot indicates one ST. The size of one spot relates to the number of pneumococcal isolates with the same ST. The lines designate the presence of single locus variant SLV and DLV links amongst STs.


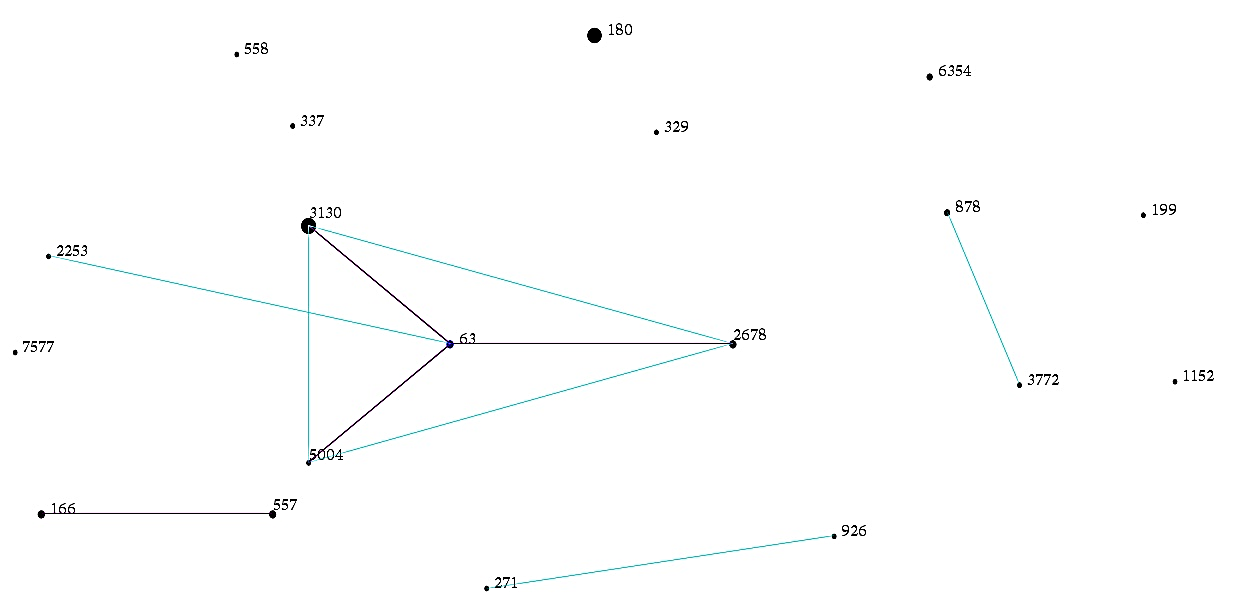

Supplement: S1 Fig — One spot indicates one ST. The size of one spot relates to the number of pneumococcal isolates with the same ST. The lines designate the presence of single locus variant SLV and DLV links amongst STs. (DOCX) [file pone.0167803.s001.docx]
