# Supplementary material for: Determination of Characteristics of Erythromycin Resistant Streptococcus pneumoniae with Preferred PCV Usage in Iran
Source: PLoS One. 2016 Dec 29;11(12):e0167803. doi: 10.1371/journal.pone.0167803 (PMC5199012; doi:10.1371/journal.pone.0167803)
Supplement: S1 Table — (DOCX) [file pone.0167803.s002.docx]

Table 1: Primers used for identification of pneumococcal isolates and ampliﬁcation of erythromycin and tetracycline resistant genes as well as transposon related genes.

| Gene^a^ | Primer sequence (5**ˈ**→3**ˈ**) | Product length (bp) |
| --- | --- | --- |
| *lytA*-F  *lytA*-R | CGGACTACCGCCTTTATATCG  GTTTCAATCGTCAAGCCGTT | 250 |
| *ply-*F  *ply-*R | ATTTCTGTAACAGCTACCAACGA  GAATTCCCTGTCTTTTCAAAGTC | 329 |
| erm(B)-F erm(B)-R | TGGTATTCCAAATGCGTAATG CTGTGGTATGGCGGGTAAGT | 745 |
| mef(A/E)-F  mef(A/E)-R | CAATATGGGCAGGGCAAG  AAGCTGTTCCAATGCTACGG | 1046 |
| TetM-F  TetM-R | GTGGACAAAGGTACAACGAG  CGGTAAAGTTCGTCACACAC |  |
| aphA3-F  aphA3-R | TAA AAG ATA CGG AAG GAA TGT CTC  TCG ACC GGA CGC AGA AGG CAA TGT | 824 |
| Int-F  Int-R | GCGTGATTGTATCTCACT  GACGCTCCTGTTGCTTCT | 193 |
| Xis-F  Xis-R | AAGCAGACTGAGATTCCTA  GCGTCCAATGTATCTATAA | 193 |
| TndX-F  TndX-R | ATG ATG GGT TGG ACA AAG A  CTT TGC TCG ATA GGC TCT A | 610 |
| TnpR-F  TnpR-R | CCA AGG AGC TAA AGA GGT CCC  GTC CCG AGT CCC ATG GAA GC | 1528 |
| TnpA-F  TnpA-F | GCT TCC ATG GGA CTC GGG AC  GCT CCC AAT TAA TAG GAG A | 2115 |
